# Effects of Electroacupuncture on Facial Nerve Function and HSV-1 DNA Quantity in HSV-1 Induced Facial Nerve Palsy Mice

**DOI:** 10.1155/2014/693783

**Published:** 2014-06-03

**Authors:** Hongzhi Tang, Shuwei Feng, Jiao Chen, Jie Yang, Mingxiao Yang, Zhendong Zhong, Ying Li, Fanrong Liang

**Affiliations:** ^1^Chengdu University of Traditional Chinese Medicine, Chengdu, Sichuan 610075, China; ^2^Sichuan Academy of Medical Sciences & Sichuan Provincial People's Hospital, Chengdu, Sichuan 610072, China

## Abstract

Acupuncture is a common and effective therapeutic method to treat facial nerve palsy (FNP). However, its underlying mechanism remains unclear. This study was aimed to investigate the effects of electroacupuncture on symptoms and content of HSV-1 DNA in FNP mice. Mice were randomized into four groups, an electroacupuncture treatment group, saline group, model animal group, and blank control group. Electroacupuncture was applied at Jiache (ST6) and Hegu (LI4) in electroacupuncture group once daily for 14 days, while electroacupuncture was not applied in model animal group. In electroacupuncture group, mice recovered more rapidly and HSV-1 DNA content also decreased more rapidly, compared with model animal group. We conclude that electroacupuncture is effective to alleviate symptoms and promote the reduction of HSV-1 in FNP.

## 1. Introduction

Peripheral facial nerve palsy (FNP) is an acute peripheral facial nerve disorder, usually affecting unilateral facial muscle, orifices, and related tissues. The clinical symptoms vary according to the location of the lesion of the facial nerve along its course to the muscles but include drooping of the brow, incomplete eyelid closure, drooping of the corner of the mouth, impaired closure of the mouth, dry eyes, hyperacusis, impaired taste, or pain around the ear [1]. An FNP patient can appear expressionless when he or she is smiling [2]. The annual incidence of FNP is estimated to be 20–25 cases per 100,000 people [3–5]. In the United States, the annual incidence was 25/100,000 people [2, 6] compared with 258/100,000 in China [7]. FNP may influence individuals of all age groups [4], with the peak incidence lying between 20 and 40 years of age. Females and males are equally affected [4] but a slight female preponderance has been observed [8]. The etiology of FNP is controversial, but viral infection, vascular ischemia, heredity, and autoimmune inflammation have been proposed as possible underlying causes [9, 10]. Evidence suggests that viral infection with herpes simplex virus type 1 (HSV-1) may predominantly occur if the immune system is compromised [4, 11–13]. Additionally, HSV-1 DNA was detected in clinical specimens, endoneurial fluid, and saliva from FNP patients [14, 15]. The herpes simplex virus (HSV) mediated viral inflammatory immune mechanism is therefore widely accepted as the major cause of FNP [14, 16].

Treatment methods of FNP are still controversial. Clinical trials investigating the efficacy of specific antiviral treatment did not show a significant benefit compared with alternative therapy [17–19]. Acupuncture is an essential part of traditional Chinese medicine (TCM), which has a history of thousands of years. Electroacupuncture is a technique combining acupuncture with electric currents. A number of studies provide evidence for a beneficial effect of acupuncture on FNP [20–22]. However, the mechanism underlying the effects of acupuncture on FNP induced by HSV-1 infection is not fully understood.

Because of difficulties in obtaining clinical specimens from patients, basic research using animal models is necessary to investigate the effects of electroacupuncture in FNP [23]. In this study, we established a mouse model of FNP induced by HSV-1 infection [24–27]. The purpose of this study was to investigate the effects of electroacupuncture on the alleviation of symptoms and content of HSV-1 in FNP mice.

## 2. Materials and Methods

### 2.1. Animals

We used 4-week-old Balb/c mice (18.5–20.5 g) purchased from Chengdu Dashuo Biological Technology Company for this experiment. All mice were maintained in Laboratory Animal Center of Chengdu University of TCM and cared for in compliance with the Guideline for Animal Experimentation at Ehime University School of Medicine.

### 2.2. Virus Inoculation and Groups

Mice were randomly divided into three groups, an FNP model group (*n* = 156), saline group (*n* = 30), and blank control group (*n* = 30). The KOS strain of HSV-1 was prepared in Vero cell sand plaque-titrated at 6.7 × 10^7^ plaque-forming units (PFU) per milliliter. Mice in the FNP model group were generally anaesthetized with intraperitoneal injection of sodium pentobarbital (50 mg/kg), and the posterior auricular branch of right facial nerve was incised by 2 mm and inoculated with 25 *μ*L virus solution (1.7 × 10^6^ PFU) on a 2 mm × 3 mm gelatin sponge, which was placed in the notch. Then, the incision was closed. In the saline group, normal saline solution was applied instead. Nothing was used on the mice in the blank control group. Animals were returned to their cages upon completion of the procedure.

### 2.3. Evaluation of FNP Model

The model was evaluated by scores of blink reflex, vibrissae movement, and position of apex nasi daily after the virus inoculation [27]. The blink reflex was evoked twice by blowing air onto the eye through an 18-gauge needle with a 5 mL syringe. The degree of blink reflex was graded on a 0 to 2 scale (0, no difference between two sides; 1, the blink reflex was delayed compared with the unaffected side; 2, the blink reflex disappeared completely). Vibrissae movement was observed for 30 s and scored on a 0 to 2 scale (0, no difference between both sides; 1, the vibrissae movement was weaker than that on the healthy side; 2, the vibrissae movement disappeared completely). The position of the apex nasi was scored on a 0 to 1 scale (0, the position is in the middle; 1: the position is to the unaffected side). The total score was defined as the sum of all scores. When the total score was 3 or 4 points, we recognized that the FNP model was established.

Only mice that developed a transient and homolateral FNP after the primary infection were used for the following experiments. Ninety mice (58%) developed FNP exclusively on the same side as the inoculation, and 60 FNP model mice were randomly divided into two groups, an electroacupuncture group (*n* = 30) and a model animal group (*n* = 30).

### 2.4. Experiment Procedures

There were four groups with 30 mice in each: Group A, blank control group; Group B, saline group; Group C, model animal group; and Group D, electroacupuncture group.

#### 2.4.1. Electroacupuncture Treatment Group

Based on previous study [22], two frequently used acupoints, Jiache (ST6) and Hegu (LI4), were selected. The location of the two acupoints was found according to* Experimental Acupuncture* [28] with both Jiache (ST6) and Hegu (LI4) on the paralyzed side. Both acupoints were punctured 3–5 mm in depth. Electroacupuncture was given by a G6805 after fixing the mice (Qingdao Xin Sheng Industrial Co., Ltd., Qingdao, China). The filiform needles were sterile Hwato acupuncture needles for single use, 25–40 mm in length and 0.30 mm in diameter (Suzhou Medical Supplies Factory Co., Ltd., Suzhou, China). The stimulation frequency was 3-4 Hz and intensity was 1-2 V in continuous wave (CW) mode to make the needle slightly vibrate and keep the mice quiet. The needles were retained for 20 min, once a day, and the treatment lasted for 14 days. Electroacupuncture practitioner in this study had 10 years of acupuncture and TCM training and 6 years of experience in academic and clinical acupuncture.

#### 2.4.2. Control Groups

There were 3 control groups, the blank control group, saline group, and model animal group. All mice were fixed once a day for 20 min without any intervention.

### 2.5. Evaluation of FNP Symptoms

After electroacupuncture at days 3, 7, and 14 of treatment period, symptoms were evaluated by scores of blink reflex, vibrissae movement, and position of apex nasi.

### 2.6. Quantification of HSV-1 DNA

HSV-1 DNA in the intratemporal facial nerve, geniculate ganglia, brainstem, and cerebral cortex tissue was quantified after electroacupuncture at days 3, 7, and 14 of treatment period.

#### 2.6.1. Extraction of HSV-1 DNA

Ten mice in each group were anesthetized by intraperitoneal injection of sodium pentobarbital (50 mg/kg) and killed by decollation quickly at days 3, 7, and 14 of treatment period. The intratemporal portions of the right facial nerves, geniculate ganglia, brainstem, and cerebral cortex were dissected and preserved at −80°C. The facial nerves, geniculate ganglia, brainstem, and cerebral cortex were cut with scissors, placed in a sample tube containing 200 *μ*L buffer solution, and shaken until they were suspended thoroughly. After removing the supernatant and adding 20 *μ*L proteinase K (20 mg/mL), the tissue was digested at 56°C for 30 min, followed by a brief centrifugation and removal of supernatant. After that, 220 *μ*L buffer solution GB was added and the sample was shaken for 15 sec and incubated at 70°C for 10 min, followed by a brief centrifugation and removal of supernatant. After adding 220 *μ*L absolute ethyl alcohol, the sample was oscillated for 15 sec and mixed thoroughly by vigorous shaking for 15 sec. Following a brief centrifugation, this solution was poured into a CB 3 absorbing column, which was put in a 2 mL collection tube and centrifuged at 9660 g for 30 sec. Waste liquid in the collection tube was removed, and the absorbing column was put back into the same collection tube. After that, 500 *μ*L deproteinized solution and 700 *μ*L and 500 *μ*L eluent GW were added consecutively and the sample was centrifuged at 9660 g for 30 sec after each addition. Waste liquid in the collection tube was removed, and the absorbing column was put back into the same collection tube and centrifuged at 9660 g for 2 min to remove residual liquid. The CB 3 absorbing column was put in a clean centrifugal tube of 1.5 mL and incubated at room temperature for 2.5 min. Buffer TE of 50 *μ*L was added to the adsorption film in the center of the absorbing column. The sample was incubated at 60°C for 2.5 min and centrifuged at 9660 g for 2 min.

#### 2.6.2. Reverse Transcription PCR of HSV-1 DNA

Synthetic primers encoding parts of the HSV-1 (5′-CCACCGAGCGGCAGGTGATC-3′ as upstream primer and 5′-GCCGACCGCCTGCTCGTGCT-3′ as downstream primer) and the *β*-actin gene (5′-CGTTGACATCCGTAAAGACCTC-3′ as upstream primer and 5′-TAGGAGCCAGGGCAGTAATCT-3′ as downstream primer) were used for PCR amplification. After an instantaneous centrifugation of primers, deionized water was added to make a 100 µM solution. Deionized water of 380 *μ*L was added in another EP tube, and 10 *μ*L upstream primers and 10 *μ*L downstream primers were added (400 *μ*L and 2.5 pmol/*μ*L). Deionized water was added to dilute cDNA to a proper concentration. The extracted DNA and primers were denatured initially. Forty cycles of amplification (15 sec at 95°C for denaturation; 15 sec at 95°C for renaturation; 45 sec at 72°C for extension) were performed and the sample was extended at 72°C for 5 min and incubated at 4°C. To test the amplification efficiency and specificity of primers, standard curves and melting curves were made. PCR solution of 24 *μ*L was made with 12.5 *μ*L SYBR Premix Ex Taq (2×), 0.5 *μ*L PCR forward primer (10 *μ*M), 0.5 *μ*L PCR reverse primer (10 *μ*M), 3 *μ*L template cDNA, and 8.5 *μ*L dH_2_O and incubated in a real-time PCR tube. Gradient dilution of 2 *μ*L for cDNA was added. Forty cycles of amplification (15 sec at 95°C for denaturation; 15 sec at 95°C for renaturation; 45 sec at 72°C for extension) were performed and the sample was extended at 72°C for 5 min. Fluorescence data for melting curve was acquired every 0.5°C during a temperature transition from 58°C to 85°C. Fluorescence quantitative standard curve was made. Real-time PCR were run in triplicate. Briefly, mRNA was corrected with *β*-actin via CT (the cycle at threshold level). The relative mRNA expression of HSV-1/*β*-actin was quantified according to the formula of 2^−ΔΔCT^. Analysis of CT was performed using Sequence Detection software version 1.2.3 (Applied Biosystems group).

### 2.7. Statistical Analysis

All analyses were carried out using the Statistical Package for the Social Sciences, version 17.0 (SPSS, Chicago, IL, USA). Descriptive statistics including the mean ± standard deviation (SD), median, minimum (min), and maximum (max) were used to present continuous variables. One-way analysis of variance (ANOVA) was used to determine statistically significant differences between groups of variables with data that were normally distributed. The least-significant difference (LSD) test was used for homogeneity of variance, whereas Tamhane's T2 test was used for heterogeneity of variance. Hierarchical data was tested with the nonparametric test to determine differences between groups. *P* < 0.05 was considered statistically significant.

## 3. Results

### 3.1. Results of Melting Curve

The HSV-1 melting curve had a single peak, melting at 85 ± 1°C. This step was replicated several times. The amplified product was the intended product.

### 3.2. Symptom Relief of Acute Viral FNP Mice

Mice in the blank control group and saline group showed no symptoms of FNP, and the score for each item in these two groups was 0. The scores of blink reflex, vibrissae movement, and position of apex nasi in the model animal group and electroacupuncture group decreased over time. However, the scores in the electroacupuncture group decreased more rapidly. The scores of blink reflex, vibrissae movement, and position of apex nasi in the electroacupuncture group were significantly lower than those in model animal group (*P* < 0.05 at day 3, *P* < 0.01 at day 7, and *P* < 0.05 at day 14 of treatment period). The differences in FNP symptoms among all groups are shown in Table 1.

### 3.3. Quantity of HSV-1 DNA

No HSV-1 DNA was detected in either saline group or blank control group mice. HSV-1 DNA was detected in the model animal group and electroacupuncture group. However, the quantity of HSV-1 was significantly lower in the electroacupuncture group compared with the model animal group (*P* < 0.05 at day 3, *P* < 0.01 at days 7 and 14 of treatment period), as shown in Table 2. At day 7 of treatment period, HSV-1 DNA was decreasing in the electroacupuncture group, while HSV-1 DNA was still higher compared with 4 days prior in the model animal group. HSV-1 DNA in the model animal group was decreasing at day 14 of treatment period.

## 4. Discussion

The blink reflex score, vibrissae movement score, position of apex nasi score, and total score were significantly higher in the electroacupuncture and model animal group than those in saline and blank control group after modeling, which indicated that the FNP mice model was successfully induced by inoculation of HSV-1. With the development of FNP, some facial nerve function recovered and HSV-1 DNA content also decreased in the model animal group. These results reflect the disease progression of FNP. Facial nerve function recovered more quickly in the electroacupuncture group. The symptom relief after acupuncture is consistent with clinical trials evaluating the therapeutic effects of acupuncture for FNP [20–22]. In addition, HSV-1 DNA quantity in the electroacupuncture group was significantly lower than that in model animal group at day 3. At day 7, HSV-1 DNA quantity in the electroacupuncture group was decreasing, while HSV-1 DNA quantity in the model animal group was still higher compared with 4 days prior. These results indicate that electroacupuncture has therapeutic effects on FNP and promotes the reduction in HSV-1.

### 4.1. The Mechanism Underlying Acupuncture to Alleviate FNP Symptoms

It has been suggested that the cell-mediated autoimmune mechanism against myelin basic protein may be the pathogenesis of FNP. FNP is an autoimmune demyelinating cranial neuritis, and, in most instances, it is a mononeuritic variant of Guillain-Barré syndrome, a neurologic disease with cell-mediated autoimmune reaction against peripheral nerve myelin antigens. In FNP, viral infection or the reactivation of a latent virus may provoke an autoimmune reaction against peripheral nerve myelin components, leading to inflammation and demyelination of the facial nerve [29]. During its progression, FNP is accompanied by significant elevations in some proinflammatory mediators, including tumor necrosis factor alpha (TNF-*α*), interleukin-6 (IL-6), and interleukin-1 beta (IL-1*β*) [30]. TNF-*α*, IL-6, and IL-1*β* are linked with a wide range of inflammatory, infectious, autoimmune, and malignant conditions [31]. The abnormal high level of serum TNF-*α* also causes demyelination of the facial nerve via inflammation [32, 33]. Acupuncture significantly reduces plasma levels of TNF-*α* in patients with chronic headache [34] and mRNA levels of IL-6 and IL-1*β* in rats of lipopolysaccharide-induced fever [35]. A recently published study found a new pathway for anti-inflammatory pathway of electroacupuncture. Electroacupuncture at the sciatic nerve controls systemic inflammation by inducing vagal activation of aromatic L-amino acid decarboxylase, leading to the production of dopamine in the adrenal medulla and then the reduction of TNF-*α* [36]. By inhibiting the production of proinflammatory mediators, acupuncture might help alleviate facial nerve inflammation and demyelination and thus promote the repair of the facial nerve to improve symptoms. Overall, acupuncture might have potential benefit for inflammation in inflammatory and infectious diseases by regulating the production of proinflammatory mediators. However, there is no direct observation to support that acupuncture could reduce the contents of TNF-*α*, IL-6, and IL-1*β* in HSV-1 induced FNP* in vivo*.

### 4.2. The Mechanism Underlying Acupuncture to Help Control HSV-1 Infection

#### 4.2.1. Acupuncture Might Help Reduce the Efficiency of HSV-1 Replication and Spread

HSV-1 can activate autoimmune pathways, including the nuclear factor kappa-light-chain-enhancer of activated B cells (NF-*κ*B) signaling pathway. Evidence suggests that HSV-1 can induce a persistent translocation of NF-*κ*B [37]. NF-*κ*B complexes are bound to inhibitors of NF-*κ*B (I*κ*Bs) in unstimulated cells, thereby maintaining NF-*κ*B in an inactive state. The phosphorylation of I*κ*B by the I*κ*B kinase (IKK) complex is involved in the activation of NF-*κ*B, leading to I*κ*B degradation, which releases NF-*κ*B and then allows it to translocate into the nucleus [38, 39]. The persistent translocation of NF-*κ*B subsequently increases the efficiency of virus replication [37]. The translocation of NF-*κ*B to the nuclei of infected cells is a necessary component to prevent apoptosis of host cells during HSV-1 infection, which helps HSV-1 evade immunological surveillance and spread effectively in the host [40]. Acupuncture can inhibit abnormal NF-*κ*B expression and activation by inhibiting the NF-*κ*B signal transduction pathway in host cells [41, 42]. This might help the immunological surveillance and reduce the efficiency of HSV-1 replication and spread. However, there is no direct observation to support that acupuncture could inhibit NF-*κ*B expression and activation in HSV-1 induced FNP* in vivo*, which needs to be further studied.

#### 4.2.2. Acupuncture Might Regulate Immune Response to Eliminate HSV-1

After HSV-1 infection, humoral immunity and cell-mediated immunity will be activated to eliminate HSV-1. In cell-mediated immunity, antigen-specific cytotoxic T lymphocytes, a type of T lymphocytes that kill cancer cells and infected cells, are activated to induce apoptosis in virus-infected cells displaying epitopes of foreign antigens on their surface. Studies regarding the effects of acupuncture on the immune system show a stimulating effect on cell-mediated immunity. Acupuncture is able to help in T lymphocyte proliferation [43]. After acupuncture, T helper lymphocytes, a type of T lymphocytes that help in the activity of other immune cells, and cytotoxic T lymphocytes were significantly increased [44, 45]. The ability of acupuncture to modulate the immune response might help the immune system to eliminate HSV-1. Therefore, acupuncture might have potential to control infection in infectious diseases by activating some immune pathways and modulating the immune response.

### 4.3. The Compatibility of Distal-Proximal Acupoints in Acupuncture Treatment for FNP

Acupoints compatibility is a key point in acupuncture prescriptions, which influences therapeutic effects. There are many classical acupoints compatibility methods, such as yuan-source acupoints and luo-collateral acupoints combination, back-shu acupoints and front-mu acupoints combination, and distal-proximal acupoints combination. Distal-proximal acupoints combination is often used in the treatment of FNP. For example, Hegu (LI4) in upper extremity and some local acupoints in face are needled in combination. Synergistic effects exist among acupoints, so acupoint compatibility can strengthen the effectiveness of acupuncture.

The mechanism underlying Hegu (LI4) treating diseases in the face and mouth remains unclear. A morphological study suggested that the Gasserian ganglion receives the neural projection to Hegu (LI4) [46]. Furthermore, the nerve in Hegu (LI4) has an indirect project to the nucleus of the solitary tract and the facial nerve has a direct connection with the nucleus of the solitary tract [47]. This might be the morphological foundation of Hegu (LI4) treating diseases in the face and mouth. As to Jiache (ST6), this acupoint is located near the subbuccal and marginal mandibular branches of the facial nerve, which provides the morphological foundation to alleviate symptoms of FNP [48]. The combination of Hegu (LI4) and Jiache (ST6) stimulates more peripheral nerves compared with needling a single acupoint of them.

## 5. Limitations

There are some limitations of this study. Although the modeling method in this study is more consistent with the natural pathogenesis of FNP, the model rate is relatively lower than that of compressing the facial nerve. Additionally, this study shows the potential of acupuncture in triggering a patient's immunoreaction to further reduce HSV-1 quantity. However, this study is insufficient to fully elucidate the mechanism of acupuncture in reducing HSV-1 quantity. Future studies might be directed toward elucidating this mechanism, such as the relationship between acupuncture and NF-*κ*B in FNP* in vivo*.

## 6. Conclusions

We concluded that, in the treatment for FNP, electroacupuncture alleviates symptoms, facilitates affected nerve recovery, and promotes the reduction in HSV-1. Acupuncture might have therapeutic advantages in controlling inflammation and infection in FNP.

## Figures and Tables

**Table 1:** Scores in evaluation of FNP during treatment period and at the end of treatment period ($\overset{-}{x}\pm S$).

Group	*N*	At day 3 of treatment period			At day 7 of treatment period			At day 14 of treatment period		
		Blink reflex	Vibrissae movement	Position of apex nasi	Blink reflex	Vibrissae movement	Position of apex nasi	Blink reflex	Vibrissae movement	Position of apex nasi
A	10	0**	0**	0**	0**	0**	0**	0**	0**	0**
B	10	0**	0**	0**	0**	0**	0**	0**	0**	0**
C	10	15 ± 3.1	17 ± 2.8	9 ± 1.8	12 ± 2.5	11 ± 1.7	7 ± 1.9	7 ± 1.8	8 ± 2.4	5 ± 1.6
D	10	7 ± 2.4*	8 ± 1.9*	3 ± 2.2*	4 ± 1.6**	5 ± 1.9**	3 ± 1.4**	1 ± 2.1*	2 ± 1.0*	0 ± 0.0*

Group A: blank control group; Group B: saline group; Group C: model animal group; Group D: electroacupuncture group. *Compared with Group C, *P* < 0.05. **Compared with Group C, *P* < 0.01.

**Table 2:** Detection of HSV-1 DNA during treatment period and at the end of treatment period ($\overset{-}{x}\pm S$).

G	*N*	At day 3 of treatment period				At day 7 of treatment period				At day 14 of treatment period			
		Facial nerve (2^−△△CT^)	Geniculate ganglion (2^−△△CT^)	Brainstem (2^−△△CT^)	Cerebral cortex (2^−△△CT^)	Facial nerve (2^−△△CT^)	Geniculate ganglion (2^−△△CT^)	Brainstem (2^−△△CT^)	Cerebral cortex (2^−△△CT^)	Facial nerve (2^−△△CT^)	Geniculate ganglion (2^−△△CT^)	Brainstem (2^−△△CT^)	Cerebral cortex (2^−△△CT^)
A	10	0**	0**	0**	0**	0**	0**	0**	0**	0**	0**	0**	0**
B	10	0**	0**	0**	0**	0**	0**	0*	0**	0**	0**	0**	0**
C	10	13.67 ± 2.16	11.82 ± 1.81	7.73 ± 1.08	6.52 ± 2.27	15.42 ± 2.71	14.18 ± 1.97	12.57 ± 3.11	6.52 ± 2.27	9.89 ± 1.47	7.63 ± 1.22	6.77 ± 1.28	6.14 ± 1.63
D	10	9.84 ± 1.29*	9.04 ± 2.29*	3.04 ± 2.29*	2.17 ± 1.14*	2.18 ± 2.13**	1.78 ± 1.14**	1.04 ± 1.48**	2.17 ± 1.25**	0.71 ± 0.16**	0.18 ± 0.21**	0.03 ± 0.09**	0.05 ± 0.13**

The quantity of HSV-1 DNA was expressed with 2^−ΔΔCT^ of difference ratio of HSV-1 mRNA. Group A: blank control group; Group B: saline group; Group C: model animal group; Group D: electroacupuncture group. *Compared with Group C, *P* < 0.05. **Compared with Group C, *P* < 0.01.
